# A novel robust evaluation approach to improve systematic behavior of failure safety in water supply system under various ellipsoid uncertainties

**DOI:** 10.1038/s41598-024-59598-z

**Published:** 2024-04-16

**Authors:** He Yuan, Moudi Mahdi, Song Xueqian, Majid Galoie

**Affiliations:** 1 College of Management, Chengdu University of Information Technology, Chengdu, 610103 China; 2 Interdisciplinary Research Center for Eco-Environmental Innovation and Governance, Chengdu, China; 3 Civil Engineering Department, Faculty of Engineering and Technology, Imam Khomeini International University, Qazvin, Iran

**Keywords:** Water shortage, Robust optimization, Failure of system, Performance of system, Water supply, Rainfall uncertainty, Climate-change adaptation, Climate-change impacts, Sustainability

## Abstract

This study proposes a novel robust optimization approach for an integrated water supply system, wherein the decision-makers attempt to improve failure safety of system under various uncertainty strategies. To cope with uncertainty, the ellipsoid uncertainty set is assumed to evaluate the best feasible solution in the direction of water supply under various strategies. We assessed the case of Hamoun watershed, a water-stressed watershed in southeastern of Iran, to evaluate the developed robust optimization model. In the following, the comparative feasibility under uncertainty levels is conducted to analyze the impacts of simulation strategies on the status of robust model. Based on the final results, the reliability of the model's objective functions experienced an increasing trend ($58.3\%$), and the objective function values under the uncertainty strategies is greatly improved. The findings of the analysis show that the robust strategies in response to the failure safety achieve outstanding optimal objectives under uncertainty.

## Introduction

The challenge of imbalance between water demand and water supply due to the impact of climate change has resulted in dissatisfaction between multiple participants and driving the performance of system toward failure^1,2^. Indeed, the performance of water supply system depends on both supply and demand sides, so that demand management postpones the need for new water resources (demand side) and, along with the optimal supply of water resources (supply side), minimizing the possibility of system failure^3,4^.

In this regard, scientific research on the failure of water supply system has recently increased due to the challenge of climatic uncertainty in the watersheds. For instance, Yan et al.^5^ proposed an integrated framework combining multi-objective robust decision-making techniques with biophysical modeling approaches to evaluate adaptive water allocation strategies that are robust to uncertainties associated with climate change. Ghelichi et al.^6^ proposed a new robust optimization approach coupled with a two-stage scenario-based stochastic programming for improving an urban water distribution system (WDS) under demand and rainfall uncertainties. The main objective is to identify solutions that are cost-efficient while satisfying both potable and non-potable water demands within an integrated system.

Lan et al.^7^ proposed a robust scenario-based optimization model to design a water supply system considering the failure risk under a set of uncertainties generated by a limited set of scenarios. Yuan et al.^8^ developed a robust optimization framework for the water-land-food nexus within agricultural irrigation systems aimed at improving the distribution of scarce water resources between multi-crops under uncertainty. Perelman and Ostfeld^9^ proposed an adjustable robust optimization (ARO) approach aimed at addressing uncertainties within the water supply system. To be specific, this method involves a dynamic decision rule policy for managing water distribution operations among conflicting sectors according to new acquired data and the evolution of the operational horizon to facilitate adaptation conditions.

Although research on robust optimization methods to enhance the water supply process is well cited in the literature, the shortage of water supply remains a primary concern within multi-sectoral systems. Most of the previous studies have pursued the desired objective functions by formulating a water supply framework, but the feasibility of water conservation policy was challenged due to the complex structure of model without considering the detailed situations. In this process, it is necessary to develop a practical method based on long-term planning, focusing on the feasibility of climate change impacts on the solution. In this regard, this study proposes a robust optimal framework for evaluating the failure safety of system on response to water conservation policy. Accordingly, the ellipsoidal uncertainty method is applied to investigate the feasibility of solution under different uncertainty strategies. This study makes the following contributions to current literature:

1. A robust optimization framework is proposed to address the water supply process while investigating the systematic behavior of failure safety between different participants.
2. Given the challenge of uncertainty in the water supply process, an ellipsoidal uncertainty model is assumed to examine the climate impacts under different strategies.

## Conceptual framework

### The developed framework for assessment of failure safety

Basically, the probability of improving the interval between water supply and water demand regarding shortage is referred to the failure of water supply system, so managing the performance-based approach can lead the system to failure safety^8–10^. In other words, performance and failure improvement are known as two components of the same discipline, so failure status is improved by optimal use of performance tools, in the sense that the high level of system performance is reflected in the prominent reduction of system failure^11,12^. In practice, the performance of water supply system includes three factors: (1) reliability "how often the system fails", (2) resilience "how quickly the system reaches a satisfactory status regarding failure occurrence", and (3) vulnerability "how prominent the probability consequences of a failure may be"^13,14^.

**Figure 1:**
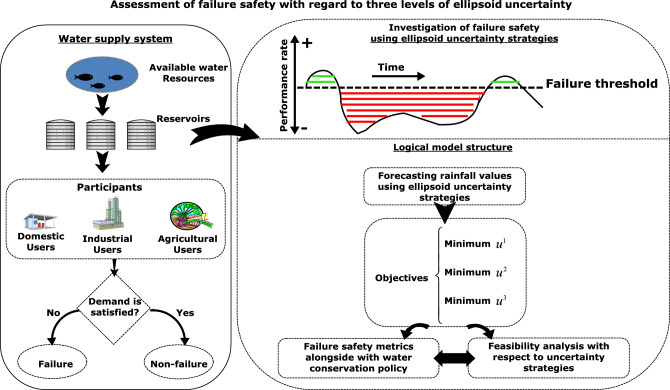
Framework of optimal water supply in terms of failure safety.

Figure 1 shows the optimal water supply to investigate the degree of system failure. Therefore, three performance indicators including reliability, vulnerability, and resilience, are taken into account to minimize objective functions in terms of failure in addition to improving water supply between multi participants. Since the uncertainty in climate patterns has a direct impact on the water allocation process, identifying the exact volume of available water is important to pursue significant outputs. In this regard, this study develops a robust optimization framework for a more flexible and feasible approach. In the following, the modeling of this study will be developed according to the provided descriptions.

The first indicator is the reliability assessment, whose inherent criteria state that an increase in reliability would reduce the failure rate of system^15,16^. The reliability of water supply system refers to the probability that the system will not fail in a given period^17,18^:

1 $$ r^{ - failure} = {\raise0.7ex\hbox{$1$} \!\mathord{\left/ {\vphantom {1 T}}\right.\kern-0pt} \!\lower0.7ex\hbox{$T$}}(\sum\limits_{t = 1}^{T} {1 - \varepsilon_{t}^{i} } )\,\,i = 1 \ldots ,n\,\& \,t = 1 \ldots ,T $$

where $T$ is referred to the total period, and $\varepsilon_{t}^{i}$ is a binary value equal to 0 and 1. If there is no failure in period $t$, demand for water is met in sector $i$, and system does not fail; so $\varepsilon_{t}^{i} = 0$. Conversely, if there is a failure, $\varepsilon_{t}^{i} = 1$.

The second criterion for failure analysis is resilience, which indicates how quickly system overcomes the failure status^19^. In fact, the resilience of the sector $i$ is the probability that the system will not experience a failure in a given time^9,20^:

2 $$ \begin{aligned} & f_{t}^{i} = \left\{ {\begin{array}{*{20}c} 1 & {if\,\eta_{t}^{i} \,UN\,failure\,\,and\,\,\eta_{t - 1}^{i} \,failure} \\ 0 & {Others} \\ \end{array} } \right. \\ & \lambda^{ - failure} = \frac{{\sum\nolimits_{t = 1}^{T} {f_{t}^{i} } }}{{T - \sum\nolimits_{t = 1}^{T} {\eta_{t}^{i} } }} \\ \end{aligned} $$

where $f_{t}^{i}$ is a binary measure with a value equal to 1 or 0, which shows a change from the failure status to the non-failure status; and $\eta_{t}^{i}$ is a projected time series of a parameter of interest.

The third measure of failure is the vulnerability, that exposes the system to failure due to the structural weaknesses^21^. However, the vulnerability of a water supply system corresponds to volumetric reliability, which examines the average severity of volumetric failure during a failure period, and indicate the magnitude of system failure due to water shortage^18,22^.

3 $$ \begin{aligned} & \delta_{t} = \sum\limits_{i = 1}^{n} {\frac{{\max (\upsilon_{t}^{i} - x_{t}^{i} ,\,\,0)}}{{\upsilon_{t}^{i} }}} \\ & \zeta^{ - failure} = \frac{1}{{T_{E}^{i} }}\sum\nolimits_{t = 1}^{T} {\frac{{\varepsilon_{t}^{i} *\delta_{t}^{i} }}{{\upsilon_{t}^{i} }}} \\ & T_{E}^{i} = \sum\nolimits_{t = 1}^{T} {\sum\nolimits_{i = 1}^{n} {\varepsilon_{t}^{i} } } \\ \end{aligned} $$

where $x_{t}^{i}$ is the amount of water allocation, and $\delta_{t}^{i}$ is the volume of water shortage, $\upsilon_{t}^{i}$ refers to the amount of water demand during period $t$ in sector $i$, and $T_{E}^{i}$ is failure index in a given time in sector $i$.

The objective functions are constraint by:

4 $$ r_{t} = r_{0} + \sum\limits_{t} {\left[ {\widetilde{{I_{t} }} - \sum\limits_{i} {x_{t}^{i} - \overline{{r_{t} }} } } \right]} \,\,,\forall t $$

5 $$ r_{t}^{\min } \le r_{t} \le \overline{{r_{t} }} \,,\forall t $$

6 $$ r_{t} \ge \sum\limits_{i} {x_{t}^{i} } \ge 0\,,\forall t $$

7 $$ \sum\limits_{t} {\sum\limits_{i} {x_{t}^{i} \le \,} } \widetilde{{I_{t} }},\forall t $$

Equation (4) depicts the amount of stored water in the reservoir in period $t$, where $\widetilde{{I_{t} }}$ is the volume of rainfall and $\overline{r}_{t}$ is referred to the maximum storage of reservoir. Equation (5) is shown that the range of available water in the reservoir must be between the minimum and maximum capacity of the reservoir. Equation (6) shows that the volume of water supplied to the participants cannot exceed the range of available water. Equation (7) indicates that the amount of water supplied to the participants continues until the divergence between water demand and water supply is higher than zero.

### Investigation of initial available water using robust optimization

As climate change uncertainty has become a major challenge in watersheds, a detailed examination of this challenge has direct implications for the water allocation process^23^. In this regard, this study develops an ellipsoidal uncertainty set to investigate the rate of initial available water. To present the modeling process, $V$ is applied as the ellipsoidal uncertainty set which can be formulated as bellow^24^:

8 $$ V = \left\{ {\overline{v}_{i} + A_{i} u\left| {\,\,\left\| u \right\|_{2} \le 1} \right.} \right\},\,i = 1 \ldots ,m $$

where $\overline{v}_{i} \in R^{n}$ and $A_{i} \in R^{n \times n}$ both refer to the inputs. However, $A_{i}$ takes a value of 0 or 1. If $A_{i} = 0$, it is needed to specify $v_{i}$, and it refers no uncertainty. Conversely, if $A_{i} = 1$, then the uncertainty set is sphere.

Accordingly, we are assumed that the independent annual values including a covariance matrix $\sum\nolimits_{IF}^{{}} {}$ as well as a nominal values vector $\beta_{IF}$ are constant during the entire period, so the second row in $\sum\nolimits_{IF}^{{}} {}$ and $\beta_{IF}$ is corresponded to the annual surface water. The ellipsoidal uncertainty sets $V$ is formulated as follows:

9 $$ V = \left\{ {\beta_{IF} + \sum\nolimits_{IF}^{1/2} {\varphi ,\left\| \varphi \right\| \le \Gamma } } \right\} $$

In Eq. (9). the perturbation vector $\varphi$ is exposed to the ellipsoid $\Gamma$ to define the tendency of decision makers to take risks, in such a way that a large value indicates stronger risk aversion and more extreme points of random vectors and a smaller value shows an optimistic trend. Thus, $\Gamma = 0$ refers to the deterministic decision. Therefore, Eqs. (4) and (7). are reformulated to robust counterpart model which is polynomially a solvable class developed by Mulvey et al.^25^.

10 $$ \left\{ {\begin{array}{*{20}l} {r_{0} + t.r_{F} \beta_{IF} + \sqrt t .\Gamma \left\| {\,r_{F}^{T} \sum\nolimits_{FR}^{1/2} {} } \right\| + \sum\limits_{t} {( - \sum\limits_{i} {x_{t}^{i} - \overline{{r_{t} }} ) \le r_{t}^{\max } ,\forall t} } } \hfill \\ { - r_{o} - t.r_{F} \beta_{IF} + \sqrt t .\Gamma \left\| {\,r_{F}^{T} \sum\nolimits_{FR}^{1/2} {} } \right\| + \sum\limits_{t} {( - \sum\limits_{i} {x_{t}^{i} - \overline{{r_{t} }} ) \le r_{t}^{\min } ,\forall t} } } \hfill \\ {\sum\limits_{t} {\sum\limits_{i} {x_{t}^{i} \le r_{F} \beta_{IF} - \Gamma \left\| {\,r_{F}^{T} \sum\nolimits_{FR}^{1/2} {} } \right\|,\forall t} } } \hfill \\ \end{array} } \right. $$

In Eq. (10). $r_{F} = (0,1)^{T}$ refers to uncertain rainfall parameter during the given period. Based on the robust optimization framework, $N$ future scenarios $\left[ {s_{t} ,t = 1,...,N} \right]$ are considered, with each scenario expressed as specific values of model parameters or time series that affect the performance of water supply system^25^. Thus, the robust optimization model is as follows:

11 $$ \begin{aligned} & \mathop {\min }\limits_{x} \left\{ {\sum\limits_{t = 1}^{T} {\varphi_{t} v_{i} (x\left| {z_{t} ),\,R\left[ {v_{i} (x\left| {z_{1} ), \ldots ,v_{i} (x\left| {z_{N} )} \right.} \right.} \right],\,\forall i} \right.} } \right\} \\ & st.\,\,\,\,\,z_{t} = M\left[ {x,\,Q(s_{t} )} \right],\,t = 1, \ldots ,N \\ & \quad g(x) \le 0 \\ \end{aligned} $$

where, $g(x)$ as the function of decision variables is considered to constrain the decisions, $v_{i} (x\left| {z_{t} )} \right.$ is referred to value of performance objective $i$ with output $z_{t}$ conditioned under scenario t, $Q(s_{t} )$ as system input refers to the rainfall parameter conditioned on scenario $s_{t}$, $R\left[ {v_{i} (x\left| {z_{1} ),...,v_{i} (x\left| {z_{N} )} \right.} \right.} \right]$ refers to robustness measure corresponding to function of objective values evaluated for $N$ scenario:$R\left[ {v_{i} (x\left| {z_{1} ),...,v_{i} (x\left| {z_{N} )} \right.} \right.} \right] = \left| {\mathop {\max }\limits_{t} v_{i} (x\left| {z_{t} ) - \mathop {\min }\limits_{t} v_{i} (x\left| {z_{t} )} \right.} \right.} \right|$, in such way that the decision maker examines the uncertain probability between the worst scenario (worst-case measure) and the best scenario (best-case measure).

To explore the feasibility of solutions, the reliability evaluation of the model $(RM)$ is applied, which refers to the ratio of the satisfaction evaluation of water supply policy corresponding to the constraints with respect to the interval between the amount of available water and water allocated to sectors $\tau = \widetilde{{I_{t} }} - \sum\limits_{i} {x_{t}^{i} }$ in all experiments ($j= 1\dots , J$):

12 $$ RM = \frac{{\sum\nolimits_{j = 1}^{J} {\mu^{j} } }}{J},\,\mu^{j} = \left\{ {\begin{array}{*{20}l} {1,} \hfill & {if\,r_{t}^{\min } \le r_{t} \le \overline{{r_{t} }} } \hfill \\ {0,} \hfill & {Others} \hfill \\ \end{array} } \right. $$

## Case study

**Figure 2:**
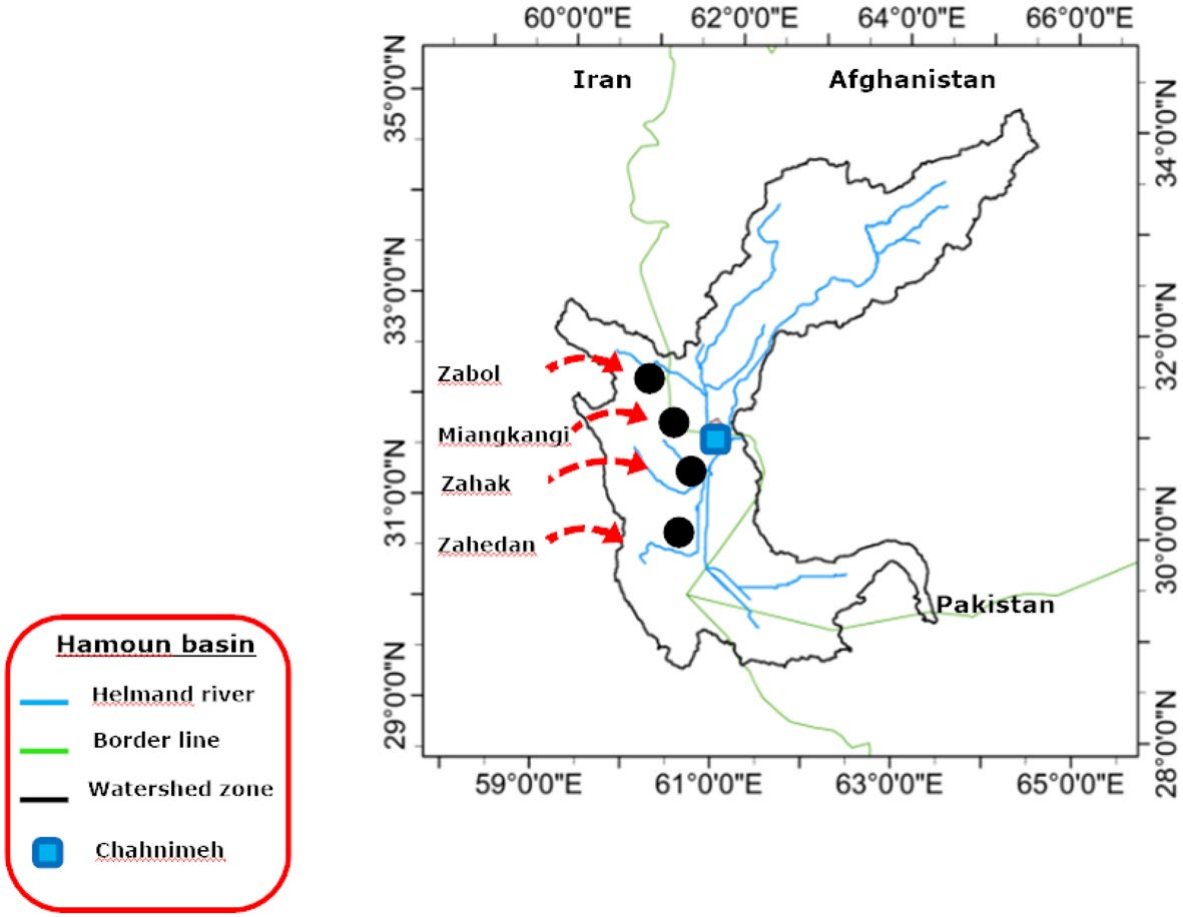
Study area, Hamoun watershed.

Hamoun watershed is fed by Helmand River located in southeastern of Iran (${30}^{\circ }$–${34}^{\circ }$ N & ${58}^{\circ }$–${64}^{\circ }$ E). Recently, the water supply to various stakeholders has experienced some challenges due to a sharp decline in the amount of water flowing into the Helmand River. Indeed, various reasons such as the decrease in seasonal rainfall, the construction of dams by Afghan government to hold back water, the increase in temperature, and the over-extraction of available water have led to diminishing available water resources^26–28^. In this regard, four large reservoirs named Chahnimeh 1, 2, 3, and 4 were built to reserve the streamflow using for different participants of Zahak, Zabol, Miangkangi, and Zahedan cities^29–31^. In the last decade, authorities have not been able to meet the water demand due to the lack of water resources, so that Hamoun watershed was chosen as the study area (Fig. 2).

The initial data for 4 sub-areas including: Zabol, Zahak, Zahedan, and Miangkangi by three sectors of domestic, industrial, and agricultural ($i$ = 3) with $\Gamma = \left\{ {0,1,2} \right\}$ over five years period ($T=5$) since 2021–2025 are considered. The random parameter $\widetilde{{I_{t} }}$ including the nominal value $\beta_{IF}$ as well as perturbation $\sum\nolimits_{IF}^{1/2} \phi$ was investigated during the given period. Indeed, $\beta_{IF}$ was extracted from the report provided by Iranian water research center as well as water resources bulletin of Regional Water Authority of Sistan and Baluchestan. The matrix $\sum\nolimits_{IF}^{1/2} {}$ was evaluated regarding Cholesky decomposition, while the covariance matrix $\sum\nolimits_{IF}^{{}} {}$ was generated based on historical data [2013–2020] obtained from the Regional Water Organization of Sistan and Baluchestan. Water demand was obtained over five years from 2021–2025, according to the data provided from statistical bulletin of Sistan and Baluchestan province as well as Iranian water research center (Table 1).

**Table 1:** Parameters of four sub-areas $(MCM)$.

${\vartheta }_{t}^{i}$	1	2	3	4	5			
Zahedan								
1	8.79	7.58	9.07	9.86	10.42			
2	14.89	15.52	16.27	14.63	17.71			
3	34.83	36.48	33.97	37.65	35.57			
Miangkangi								
1	3.21	3.68	4.82	3.99	4.36			
2	7.79	7.25	8.01	9.54	7.68			
3	51.64	52.39	52.89	54.28	53.19			
Zabol								
1	5.98	6.37	6.97	7.26	8.12			
2	9.51	10.14	11.34	14.87	13.45			
3	48.37	50.13	51.34	51.97	53.01			
Zahak								
1	5.02	4.58	5.72	5.19	5.96			
2	9.17	8.80	8.52	9.35	9.73			
3	54.98	56.07	55.45	56.71	56.29			

	Ch1	Ch2	Ch3	Ch4	Zahedan	Miangkangi	Zabol	Zahak
Dead storage	48.54	42.27	46.84	87.18	21.15	14.69	18.27	17.63
Active storage	144.35	110.71	134.85	385.95	57.98	28.75	48.39	40.00
Total storage	220.00	220.00	220.00	820.00	150.00	100.00	150.00	120.00
Year	2013	2014	2015	2016	2017	2018	2019	2020
Rainfall (${{\text{m}}}^{3}{{\text{s}}}^{-1})$	5.87	5.26	6.72	5.43	6.12	6.70	6.25	5.92

Participants: 1: domestic sector, 2: industrial sector, 3: agricultural sector.

Chahnimeh1: Ch1, Chahnimeh2: Ch2, Chahnimeh3: Ch3, Chahnimeh4: Ch4.

*Dead storage* is referred to the minimum capacity of the reservoir, *Active storage* is referred to live capacity of the reservoir,

Total storage is maximum capacity of reservoir.

## Results and analysis

### Trade-off between ${\varvec{R}}{\varvec{M}}$, objectives, and solution

Three robust strategies including $RC1, RC2,$ and $RC3$ are considered in terms of the degree of uncertainty $\Gamma = \left\{ {0,1,2} \right\}$. To investigate the performance of optimal water supply planning and improved failure, a simulation with 1000 random samples generated by the uncertainty set $V = \left\{ {\beta_{IF} + \sum\nolimits_{IF}^{1/2} {\varphi ,\left\| \varphi \right\| \le \Gamma ,\Gamma = \sqrt 5 } } \right\}$ was considered. Also, evaluating the gap between the amount of available water and the volume of water allocated ($\tau$) in the simulation experiments was taken into account.

**Figure 3:**
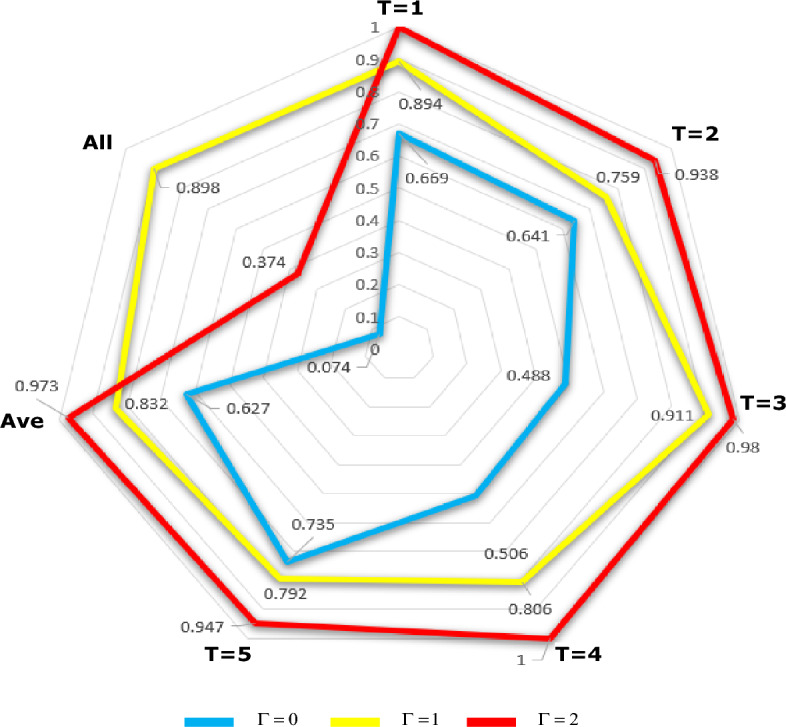
Investigating the reliability of optimal solution for water supply process.

Figure 3 shows the reliability output under available water constraints. The output figures differ significantly among the three experiments $\Gamma = \left\{ {0,1,2} \right\}$ with respect to the availability of water resources and $\tau$. The uncertainty range of the feasibility status in 1000 samples acquired an average water storage of $62.69\%$ for $RC1$, with $83.28\%$ for $RC2$, and $RC3$ of 97.27%. Since all figures must comply with the operating limits simultaneously, the figures for the entire period reach a lower value. Meanwhile, the values of reliability for the whole period could not be valid, so there was a significant difference in the overall reliability. Accordingly, the overall water storage achieved a feasibility of $89.8\%$ for the $RC2$ strategy, while the $RC1$ strategy recorded an infallibility of $93.6\%,$ and the value of $RC3$ reached $37.4\%$. Although $RC1$ and $RC3$ strategies violated almost all constraints, feasibility of $RC2$ was prevailed in most cases.

Table 2 shows the optimal solution of water supply along with the correlation rate between $RM$ and $\tau$ values. The rate of ${RM}_{c}$ recorded a value of about $18.73\%$ (for the $RC1$ strategy) to $82.70\%$ (for the $RC3$ strategy) per overall period, an increase of $58.3\%$ in the objective values ${RM}_{o}$, and an average increase of $3.26 (MCM)$ in the rate of water supplied. Indeed, the $RC3$ strategy improved the $RC1$ strategy in terms of ${RM}_{c}$ and ${RM}_{o}$. While the optimal decision-making of a deterministic in uncertain environment leads to non-adaptation, robust strategies achieve optimal adaptive solutions of resources under uncertainty with low possibility of failure.

**Table 2:** Evaluation of constraint reliability ${RM}_{c} \left(\%\right)$ as well as objective reliability ${RM}_{o}$ $(\%)$ along with $\tau (MCM)$ in terms of solutions for water supply process.

Strategy	1	2	3	4	5
$${\varvec{R}}{\varvec{C}}1$$					
${RM}_{c}$	18.73	17.27	19.89	20.04	23.67
${RM}_{o}$	16.31	19.87	17.50	16.18	19.42
$\tau$	7.73	6.53	5.84	4.36	5.75
$${\varvec{R}}{\varvec{C}}2$$					
${RM}_{c}$	35.19	38.52	40.00	45.02	52.33
${RM}_{o}$	31.11	33.82	34.65	38.26	40.57
$\tau$	9.95	8.22	7.38	6.24	6.02
$${\varvec{R}}{\varvec{C}}3$$					
${RM}_{c}$	66.47	68.29	73.51	79.34	82.70
${RM}_{o}$	66.98	67.30	69.95	73.26	74.89
$\tau$	11.08	10.23	9.87	10.57	8.13

### Optimal outputs of water supply with respect to conservation policy

**Figure 4:**
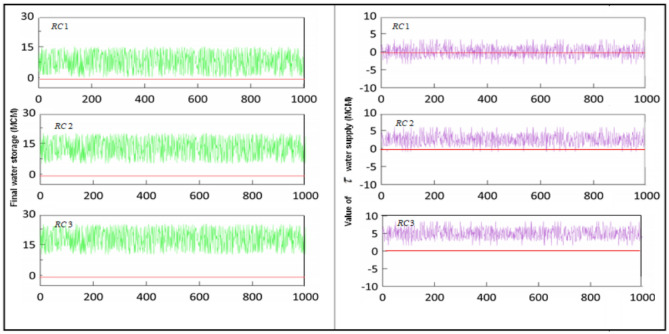
Simulation outputs of water conservation and $\tau$ value regarding three strategies.

Figure 4 illustrates the volume of water conservation and $\tau$ are significantly influenced by uncertain strategies. Based on the final outputs, $RC1$ has fluctuated between $2.89$ and $14.73$ ($MCM$); $RC2$ has varied from $6.95$ to $17.688 (MCM),$ and $RC3$ has changed from $9.85$ to $23.37 (MCM)$, while only $186$ infeasible values in 5*1000 samples were identified.

The rate of $\tau$ experienced a trend similar to water conservation fluctuations. $RC1$ recorded the worst situation with a minimum value equal to $-2.41 (MCM)$ and a maximum value of $3.97 (MCM)$, $RC2$ obtained a more optimal value compared to the previous strategy $[0.33, 6.02] (MCM)$ which is located in the set, and the most stable output was assigned to the $RC3$ strategy with a value between $3.18$ and $7.36 (MCM)$. Evaluating the outputs shows that almost all the values $(94\%)$ have acquired the feasible states to satisfy the conditional constraints. In general, the simulation strategies show that the optimal solutions in terms of deterministic programming framework lead to undesirable outputs, while the development of robust strategies achieves reasonably satisfactory outputs.

**Figure 5:**
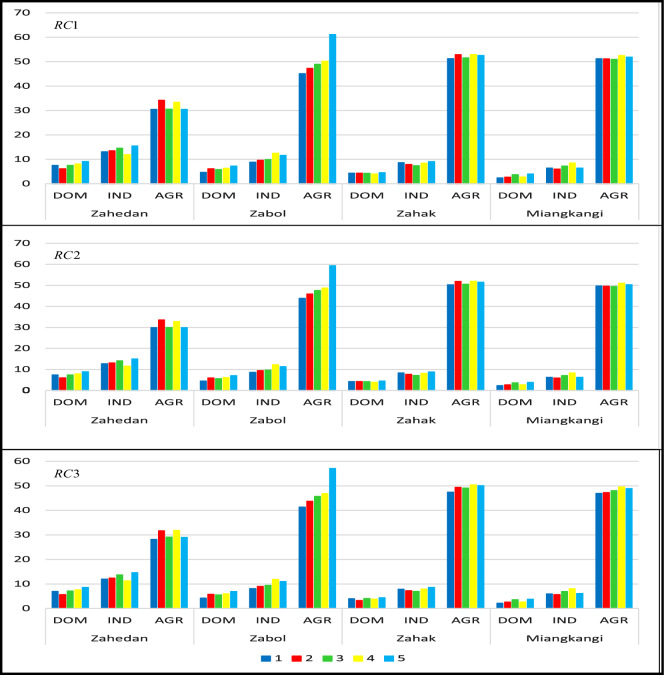
Simulation of optimal water supply $(MCM)$ under various strategies.

Figure 5 plots the optimal regional water supply strategy between the four cities of Zahedan, Zabol, Zahak, and Miangkangi, conducting three experiments of $RC1, RC2,$ and $RC3$ in response to the uncertainty level of rainfall $I$. By adopting the optimal model under the three aforementioned strategists, the total amount of water supplied gradually decreased, as the conservative optimal scheme was developed in response to the shrinkage of water resources. In terms of water scarcity, agricultural irrigation is the first sector to be experienced a reduction in water intake because its consumption is significantly higher than that of the other two sectors, conserving the irrigation water. By adopting the optimal regional water allocation strategy, Zabol has received the most water with an average of $62.49 (MCM)$, since the water demand for irrigation (farming is the main occupation of the local people) in this sub-area is higher than that of the other sub-areas. Although Zahedan has a larger population compared to other cities, the average volume of water withdrawal $(49.88 (MCM))$ was much lower than Zabol. The average volume of water supplied to other two regions (Zahak and Miangkangi) was equal to $60.86 (MCM)$ and $57.58 (MCM)$, respectively.

In addition, evaluating the optimal objective outputs regarding various uncertainty levels indicates that the increase in the value $\Gamma$ has led to improved objectives in most sectors (Table 3). Comparing the figures of ${u}^{1}$ and ${u}^{2}$ under $RC2$ and $RC3$ strategies, we find a significant divergence of about 0.3 between the agricultural sector and the domestic sector, indicating the agricultural sector is the most sensitive to extreme uncertainty in terms of ${u}^{1}$ and ${u}^{2}$. Conversely, the industrial sector reached the highest values in terms of ${u}^{3}$, implying that this sector is more sensitive to extreme hydrological uncertainties. According to the final outputs, it can be argued that the industrial and the agricultural sectors are the major source of failure in water supply system. In all, Miangkangi has acquired the more optimal figures in all objective functions due to less water intake, resulting in more failure safety.

**Table 3:** Reflection of various strategies on objective functions.

	Participant	Strategy	${u}^{1}$	${u}^{2}$	${u}^{3}$
Zahedan	1	$RC1$	0.30	1	0.0157
		$RC2$	0.33	0.33	0.0187
		$RC3$	0.32	0.29	0.0163
	2	$RC1$	0.47	1	0.0179
		*RC2*	0.41	0.33	0.0137
		*RC3*	0.42	1	0.0198
	3	*RC1*	0.50	0.38	0.0121
		*RC2*	0.55	0.43	0.0167
		*RC3*	0.50	0.36	0.0188
Zabol	1	*RC1*	0.31	1	0.0137
		*RC2*	0.29	0.22	0.0158
		*RC3*	0.24	0.26	0.0106
	2	*RC1*	0.39	1	0.0164
		*RC2*	0.37	0.36	0.0186
		*RC3*	0.32	0.35	0.0197
	3	*RC1*	0.45	1	0.0119
		*RC2*	0.39	0.51	0.0133
		*RC3*	0.52	0.34	0.0168
Zahak	1	*RC1*	0.25	1	0.0110
		*RC2*	0.23	0.27	0.0152
		*RC3*	0.26	0.20	0.0145
	2	*RC1*	0.35	1	0.0184
		*RC2*	0.33	0.29	0.0152
		*RC3*	0.30	0.32	0.0169
	3	*RC1*	0.47	1	0.0123
		*RC2*	0.45	0.55	0.0147
		*RC3*	0.52	0.49	0.0111
Miangkangi	1	*RC1*	0.28	1	0.0168
		*RC2*	0.24	0.21	0.0134
		*RC3*	0.22	0.19	0.0173
	2	*RC1*	0.37	1	0.0184
		*RC2*	0.33	0.32	0.0183
		*RC3*	0.39	0.27	0.0172
	3	*RC1*	0.49	1	0.0107
		*RC2*	0.57	0.38	0.0113
		*RC3*	0.58	0.39	0.0159

### Feasibility analysis of model under deterministic strategy

The proposed model of this study considered three different strategies $\Gamma = \left\{ {0,1,2} \right\}$ regarding the level of uncertainty. According to the results, the changes in the level of uncertainty caused prominent differences in the failure safety objectives and the rate of water supplied to participants under conservative policy. In general, the value of optimal water supply is significantly different from the value of ${u}^{1}$, ${u}^{2}$, and ${u}^{3}$ under deterministic strategy ($RC1$) compared to the other strategies ($RC2\&RC3$). Table 3 shows that the deterministic strategy ($RC1$) obtained the best optimal water supply values with a minimum water deficit of $4.36 (MCM)$ and a maximum rate of $7.73 (MCM)$, while the minimum shortage for robust strategies of $RC2$ and $RC3$ were $6.02$ and $8.13 (MCM),$ which raised by $3.19\mathrm{ \%}$ and $6.04\mathrm{ \%},$ respectively. It must be mentioned that the value of ${u}^{2}$ under $RC1$ was not changed as the state of system was not altered from failure to non-failure. Conversely, the values of ${u}^{1}$, ${u}^{2}$, and ${u}^{3}$ under strategies $RC2$ and $RC3$ have been greatly improved compared to the deterministic strategy ($RC1$), resulting improved failure safety of system.

### Discussion

Considering the challenge of failure in water supply systems, developing new strategies in response to various levels of uncertainty has influenced the balance between water supply and water demand, leading to a significant improvement in failure safety. Following the preceding analysis, some highlights must be considered:

## Highlight 1: Adopting water conservation policies and some adaptation strategies

According to the findings, the enhancement of system failure was most significantly influenced by the factors of vulnerability and reliability. The agricultural and industrial sectors, as the largest recipients of water resources, exhibited the highest sensitivity to vulnerability and the lowest sensitivity to reliability. Consequently, these sectors had the most substantial influence on system failure and contributed to reduced stability. The decision-makers in the aforementioned sectors primarily prioritize economic benefit, regardless of the varying levels of water consumption within each sector. Therefore, given the paramount importance assigned to the conservation of water resources, there is a notable emphasis on reevaluating decision-making strategies concerning the anticipated challenges associated with the degradation of water resources. The implementation of adaptation strategies such as recycling wastewater for use in industrial and agricultural sectors, adopting drip irrigation over furrow irrigation, altering cultivation methods, and exploring desalination represents a significant step toward offering a viable and practical alternative. These measures are intended to enhance the conservation of water resources, thereby mitigating the influence of shortage.

## Highlight 2: Adjusting water supply systems to accommodate extreme inflow scenarios

Failing to alter the resilience level of the system under various scenarios signifies maintaining the system's failure state rather than transitioning it to a non-failure state. This practically indicates an inability to meet the water required by participants due to scarcity. As drought is an enduring phenomenon resistant to elimination, efforts are highlighted towards mitigating its impacts. The presence of diverse stakeholders with varying demands and priorities within a water supply system, compounded by climatic uncertainty, highlights the limited efficacy of conventional water resource management in significantly enhancing failure safety. Consequently, improving overall resilience to stress necessitates the adoption of flexible decision-making approaches. Emphasizing only the supply aspect while neglecting the demand component leads to the inefficient utilization of scarce water resources. To facilitate the development of resilience in optimal water supply models and improve failure safety, it is imperative to forecast spatial–temporal patterns of population density and anticipate modifications in land use over the forthcoming years. This proactive approach is essential for effectively managing the demands of stakeholders.

## Conclusion

The water allocation model was investigated to track the failure safety of water supply system between stakeholders in a watershed with multi-year stress under uncertainty strategies. The main points reflected from the water withdrawal allocation system include: 1) minimizing the mismatch between demand and supply parameters in response to water conservation policy to improve the failure safety of system; 2) different strategies pursue the performance and feasibility of the model under different levels of uncertainty. Indeed, evaluating the RO models based on the ellipsoid uncertainty set reveals the uncertainty of the elasticity of the models considering surface waters. The outputs represent a trade-off between objective functions and reliability with regard to the various protection levels. The Hamoun watershed, a water-stressed watershed in southeastern of Iran, was considered as the study area to evaluate the feasibility of optimal framework. The optimal outputs of $RC$ strategies with different levels of uncertainty were investigated and various scenarios including deterministic and no-deterministic uncertainty were also compared. Finally, the proposed framework can be developed in terms of underground water resources as well as desalination, the uncertainty of demand, and the market-based trends between participants.

## Data Availability

All data generated or analyzed during this study are included in this published article.
